# *Aegle marmelos* (L.) Leaf Extract Improves Symptoms of Memory Loss Induced by Scopolamine in Rats

**DOI:** 10.3390/foods13040627

**Published:** 2024-02-19

**Authors:** Chanida Thongsopha, Thanasit Chaiwut, Pornnarez Thaweekhotr, Paiwan Sudwan, Noppadol Phasukdee, Ranida Quiggins

**Affiliations:** 1 The Department of Anatomy, Faculty of Medicine, Chiang Mai University, Chiang Mai 50200, Thailand; chanida_tho@cmu.ac.th (C.T.); paiwan.sudwan@cmu.ac.th (P.S.); noppadol.phasukdee@gmail.com (N.P.); 2 Graduate School, Chiang Mai University, Chiang Mai 50200, Thailand; 3 The Department of General Education, Kanchanabhishek Institute of medical and Public Health Technology, Nonthaburi 11150, Thailand; thanasit.ch@kmpht.ac.th; 4 The School of Integrative Medicine, Mae Fah Luang University, Chiang Rai 57100, Thailand; pornnarez.tha@mfu.ac.th

**Keywords:** Alzheimer’s disease, *Aegle marmelos*, memory test, synaptophysin, dendritic spine

## Abstract

Alzheimer’s disease (AD) is the most common neurodegenerative disease that results in memory impairment. *Aegle marmelos* (L.) Correa (*AM*) is used as a traditional medicine. *AM* leaves have the potential to inhibit acetylcholinesterase activity. This study used scopolamine to induce AD in rats. The aim of this study was to investigate the effects of *AM* leaf extract using this model. Motor and memory functions were tested by the motor activity and Morris water maze (MWM) tests, respectively. The density of the synaptophysin and dendritic spines in the CA1 were detected by immunofluorescence and Golgi impregnation, respectively. The hippocampal histology was reviewed by H&E staining. After the treatment, the latency times in the MWM tests of the AD groups reduced, while the motor activities showed no difference. The density of the synaptophysin of the AD groups increased after the treatments, and that of the dendritic spines also increased in all AD groups post-treatment. The hippocampal tissue also recovered. *AM* leaf extract can improve cognitive impairment in AD models by maintaining the presynaptic vesicle proteins and dendritic spines in a dose-dependent manner.

## 1. Introduction

Alzheimer’s disease (AD) is the most common neurodegenerative disease. It is found in people aged 65 years or older [1,2] and is the most common cause of dementia. The most significant symptoms include a reduced ability to learn, cognitive problems, and memory dysfunction. In Alzheimer’s disease, the neurons involved in cognitive impairment are damaged and degenerated [3]. These pathological conditions result in reduced neurotransmitters in cholinergic neurons [1,3,4,5]. Normally, cholinergic neurons are responsible for releasing acetylcholine (ACh) in the peripheral and central nervous systems. There are two subtype receptors: nicotinic acetylcholine receptors (nAChR) and muscarinic acetylcholine receptors (mAChR) [5,6]. Cholinergic neurons are mostly located in the nucleus basalis of Meynert (nbM) and the septal nuclei in the basal forebrain. These neurons send their projections to the cerebral cortex and hippocampal formation, which is associated with memory functioning in AD [3,5].

Currently, scopolamine is used as the standard drug to induce AD-like diseases or dementia in animal models. Scopolamine is a nonselective muscarinic receptor antagonist and has the structure of an acetylcholine neurotransmitter [7]. Scopolamine has been found to cause cholinergic dysfunction, which consequently causes cognition impairments and oxidative stress in the brains of rats [8]. Memory impairment occurs as a result of increased oxidative stress in the brain due to changing levels of antioxidant enzymes, combined with increasing levels of Ach in the cortex and hippocampus [9]. Scopolamine induces atrophy of neuronal cells and causes degenerative changes in the hippocampus [10]. Vacuolations within the surrounding neuropil have been demonstrated in the hippocampi of animals treated with scopolamine [11]. The density of the dendritic spines of neurons in the CA1 region of the hippocampus decreased in these animals, as revealed via the Golgi impregnation method [12]. In addition, an immunofluorescence assay demonstrated that the level of synaptic proteins, such as Syntaxin, PSD-95, and SNAPS-23, decreased in the cortex, the CA1, and the dentate gyrus of scopolamine-treated mouse models, causing synaptic dysfunction and leading to memory problems [13].

Acetylcholinesterase inhibitors (AChEIs) in medical form are currently used to treat AD patients and are approved by the US Food and Drug Administration (FDA). AChEIs increase the ACh level in the synaptic cleft of cholinergic neurons [5,14,15]. However, many studies have examined natural products that might improve learning and memory, as well as the pathology of AD [16,17]. Herbal medicines have various bioactive compounds that have been shown to benefit AD sufferers, as they are anti-inflammatories, antioxidants, and anti-amnesics [17,18]. *Aegle marmelos* (L.) Correa (*AM*) is commonly known as Bael, Bengal quince, golden apple, or stone apple. Bael fruits are used to make tea, syrup, and desserts, and are one of the ingredients found in bakery cakes [19]. *AM* leaves are also used as a Thai herb. There are many pharmacological properties of *AM*, including anti-inflammatory, antioxidant, anti-amnesic, memory-enhancing, and anti-acetylcholinesterase properties [20,21,22,23]. *AM* can reduce oxidative stress, memory impairment, neuroinflammation, and cholinergic hypofunction in the hippocampal area of AD rats [24]. AD models treated with *AM* extract have demonstrated decreasing latency times and increased time spent in the target quadrant when undergoing a Morris water maze (MWM) test [20,24]. Pre-treatment with *AM* has demonstrated a significant improvement in the size and shape of neurons, without any neuronal shrinkage in the hippocampal region of AD rats, along with preventive effects for neuronal tissue in the striatum, cerebellum, and cerebrum [24,25]. Phytochemical analysis of an ethanolic extract of *AM* leaves has revealed that they consist of alkaloids, carbohydrates, phenols, tannins, flavonoids, and terpenoids [26]. The phytochemical compounds isolated from the leaves include skimmianine, aeglin, rutin, Y-sitosterol, β-sitosterol, flavone, lupeol, cineol, citral, glycoside, and O-isopentenyl [27]. The objective of this study was to determine the therapeutic effects of *AM* leaf extract in scopolamine-induced cognitive impairment and neuronal damage.

## 2. Materials and Methods

### 2.1. Aegle marmelos (L.) Correa (AM) Leaf Preparation and Extraction

Fresh mature *AM* leaves were used to make herbarium specimens (QBG. No. 122080) and were authenticated by the Queen Sirikit Botanic Garden Herbarium, Ministry of Natural Resources and Environment. The leaves were collected and dried in a hot air oven at 50–55 °C, ground to a powder, and then soaked in absolute ethanol for 7 days. The *AM* leaf extract was filtered using Whatman No. 1 filter paper, the ethanol was evaporated by rotary evaporator, and the extract was then freeze-dried using an LTE freeze dryer and stored at 4 °C. It was then dissolved in 10% Tween 80 before being used for intraperitoneal administration.

### 2.2. Aegle marmelos (L.) High Performance Liquid Chromatograph (HPLC)

Phytochemicals of *AM* extract were analyzed by HPLC-diode array. The HPLC was performed using an Agilent 1260 Infinity Binary LC (Santa Clara, CA, USA). The chromatographic conditions were based on a previous study [28]. The *AM* extract was separated on a 150 mm × 4.60 mm Purospher^®^ Star PR-18 end capped column with a particle size of 5 µm. The mobile phase consisted of water containing 0.1% formic acid and acetonitrile (92:8 % *v*/*v*) at a flow rate of 0.8 mL/min set for 10 min. The injection volume of sample was 10 µL, and acetonitrile was increased as described here: time (min)/% acetonitrile 24/14, 35/23, and 60/24, respectively. The detection wavelengths were set at 250 nm and 330 nm. Spectra between 200 nm and 400 nm were collected for analysis. Peak identification in the chromatogram was performed by comparing retention times and spectral characteristics with the standard.

### 2.3. Animals and the Experimental Protocol

**Figure 1:**
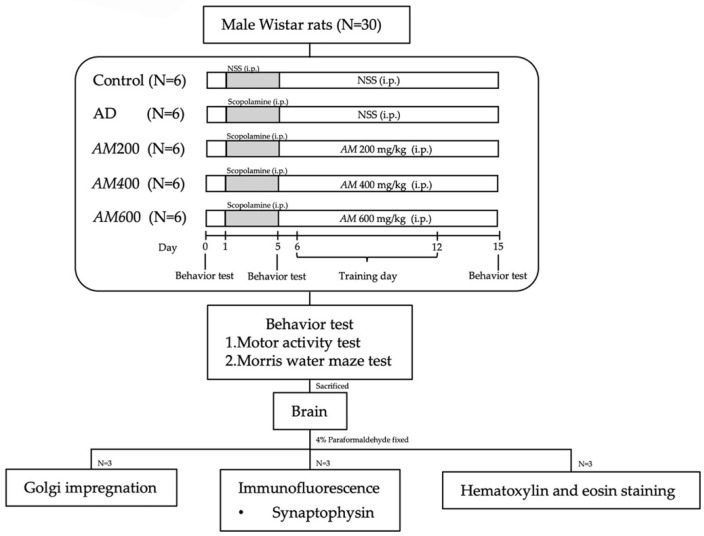
Experimental protocol for 15 days. The rats were divided into 5 groups (*n* = 6). The control group received 0.9% NSS. The AD group received 1 mg/kg BW of scopolamine for 5 days. The *AM*200 group received *AM* leaf extract at a dose of 200 mg/kg BW. The *AM*400 group received *AM* leaf extract at a dose of 400 mg/kg BW. The *AM*600 group received *AM* leaf extract at a dose of 600 mg/kg BW. Motor activity and MWM tests were conducted on days 0, 5, and 15. The rats were trained on the MWM test on days 6–12. On day 16, the rats’ brains were removed. MWM: Morris water maze.

A total of 30 male albino Wistar rats, aged 6 weeks and weighing 150–200 g (BrlHan: WIST@Jcl (GALAS), Nomura Siam International, Bangkok, Thailand) were caged at room temperature (25 °C) with a 12/12-h light/dark cycle and were provided with food and water ad libitum. The experimental protocol was approved by the Animal Ethic Committee of the Faculty of Medicine, Chiang Mai University (approved protocol no., 27/2562). The rats were randomly divided into five groups (six rats per group). The control group received intraperitoneal (i.p.) injections of 1 mL of saline for 15 days. The scopolamine (or AD) group and the other three *AM* groups received 1 mg/kg of scopolamine (Sigma, Lot no. LRAB7821, St. Louis, MO, USA) for 5 days. Next, the *AM*200 [20,24], *AM*400 [20,24], and *AM*600 groups were treated with the *AM* leaf extract for 10 days at doses of 200, 400, and 600 mg/kg of body weight, respectively. All animals were tested for their motor and memory behaviors and their hippocampi were investigated for synaptophysin, dendritic spine, and histological features. All the protocols and experiments in this study are shown in Figure 1.

### 2.4. Motor Activity Test

Motor activity was estimated in an automated frame box (25 × 25 cm frame) containing an infrared beam that determined the frequency of movement to monitor motor activity (LE8825, PANLAB, Barcelona, Spain). Before the trial, 30 min were allocated to allow the rats to rest. The tested rats were put into the frame box for 5 min, where the frequencies of their movements were detected by beams connected to a computer analyzer.

### 2.5. Morris Water Maze (MWM) Test

The MWM test consists of a circular water pool with a 110 cm diameter and a 43 cm depth, as well as a round platform of 10 cm in diameter and 29 cm in height placed in one of the four quadrants (N, S, E, and W) at 1 cm below the water surface. The water that filled the pool was 25 ± 1 °C. The rats had been trained to find the submerged platform in 4 MWM test trials performed on days 0 and 5 and then 4 trials per day for 7 days (days 6–12) before the final MWM test performed on day 15. The rats had 60 s to find the platform and 30 s to rest on the platform. The latency time or the time it took the rats to find the platform was observed in every trial. The MWM test was performed on days 0, 5, and 15.

### 2.6. Animal Perfusion and Brain Sectioning

On day 16, the rats were anesthetized with an overdose of isoflurane and were perfused transcardially with a 0.1 M phosphate buffer (PB) followed by 4% paraformaldehyde fixatives. Their brains were then removed. Three brains from each group were sectioned into 25 μm thicknesses from the rostral of the caudal sections using a vibratome (Leica s1200, Tokyo, Japan). Every fifth section was collected and immersed in 0.1 M PB.

### 2.7. Immunofluorescence and Analysis of the Synaptophysin Density

The brain sections containing the hippocampus were incubated in 3% hydrogen peroxide overnight and then were rinsed in 0.1 PB 3 times for 10 min. After blocking in 10% normal goat serum (NGS), 0.5% Triton X-100, and 0.1 M phosphate-buffered saline (PBS) for 2 h at room temperature, the brain sections were incubated in a 1:1000 dilution of anti-synaptophysin made in rabbits (Sigma, Lot no. 310333) diluted into 1% NGS in PBS at 4 °C overnight, followed by 1:100 goat anti-rabbit antibody-conjugated Rhodamine diluted in PBS (Millipore lot no. 2775066, Burlington, MA, USA) for 1 h. The sections were counter-stained with 1 μg/mL of DAPI and then washed in PB. The synaptophysin-labeled presynaptic vesicle proteins in the CA1 of the hippocampus were observed under a fluorescent microscope (Zeiss Axio Scope. A1, Tokyo, Japan) and were photographed using the 20× objective. The density of the synaptophysin was analyzed using the ImageJ program (https://imagej.net/ij/). The grid in which 1000 µm^2^ square areas were laid over the synaptophysin labeling area, and 10 of these square areas were systematically selected for counting and analyzing the synaptophysin density. These data were compared to those of the five animal groups.

### 2.8. Golgi Impregnation and Analysis of Dendritic Spine Density

Three rat brains containing the hippocampus from each group were cut into approximately 5 × 5 × 5 mm^3^ blocks. These blocks of brain tissue were immersed in 2% potassium dichromate for 3 days in the dark. After rinsing with distilled water, they were immersed in 1% silver nitrate and slowly shaken for 3 days in the dark, and then rinsed with distilled water [29]. The tissue blocks were coronally cut into 30 μm thicknesses using a vibratome (Leica s1200) and were collected in 0.1 M PB. Lastly, the sections were put onto a slide, dehydrated with ethanol followed by xylene, and the coverslip was mounted. The impregnated dendritic spines of the neurons in the CA1 area were observed under a light microscope (Nikon Eclipse E200, Tokyo, Japan). A total of 150 impregnated secondary dendrites per group (50 secondary dendrites/case) were photographed using the 100× objective and were analyzed using the ImageJ program. The number of dendritic spines per 10 µm length is reported as the dendritic spine density [30,31].

### 2.9. Hematoxylin and Eosin Staining

One row of the brain sections was selected. The sections were stained in hematoxylin for 5 min and then rinsed three times in tap water, followed by a bluing reagent. After rinsing, the sections were stained in eosin for 10 min and then dehydrated through two changes of 95% and 100% of ethanol. The sections were cleared with xylene and covered by coverslips. The H&E-stained hippocampi were examined under a light microscope (Nikon Eclipse E200). The CA1, CA2, and CA3 areas and the dentate gyrus were photographed using the 20× objective.

### 2.10. Statistical Analysis

The data are presented as the mean ± SD. One-way analysis of variances (ANOVA) was used to analyze the differences between groups in SPSS version 22, followed by Tukey’s post hoc test. The significance level was considered to be *p* < 0.05.

## 3. Results

### 3.1. High-Performance Liquid Chromatography (HPLC) Analysis

**Figure 2:**
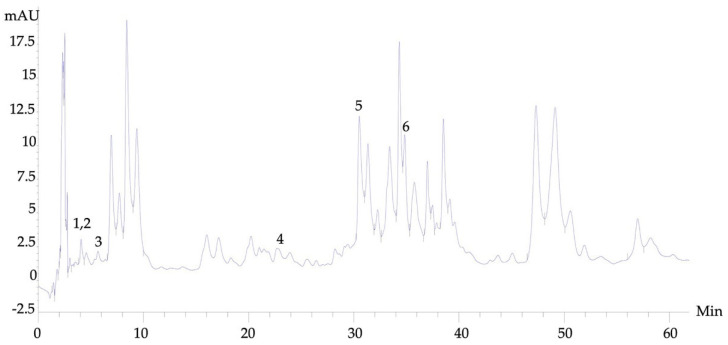
High–Performance Liquid Chromatography profiles of *AM* extract. Peak 1–6 are cumin aldehyde, gallic acid, euginol, caffeic acid, flavone, and rutin, respectively.

There were several phytochemical compounds in the AM extract that were identified by HPLC. Cumin aldehyde, gallic acid, euqinol, caffeic acid, flavone, and rutin were found in the AM extracts, and the retention times were 4.054, 4.066, 6.967, 22.787, 30.56, and 34.62 min, respectively (Figure 2). Flavone and rutin were the two most abundant contents in the AM extract.

### 3.2. Effects of the AM Leaf Extract on Motor Activity

**Figure 3:**
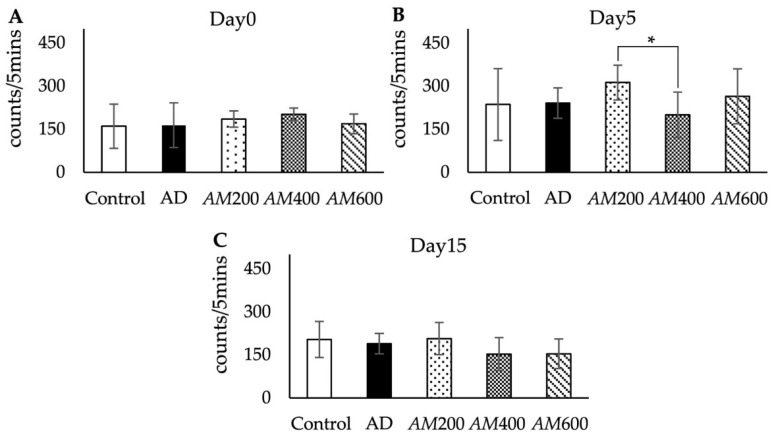
Histograms showing no significant differences in the motor activity among the groups on day 0 (the day before induction; (**A**)) and day 15 (10 days after *AM* treatment; (**C**)). On day 5 (5 days after induction; (**B**)), the motor activity of the *AM*200 group showed a significant difference from that of the *AM*400 group. The data are shown as the mean ± SD; (*n* = 6), * *p* < 0.05, analyzed via a one-way ANOVA. AD: Alzheimer’s disease-like symptoms; *AM*200: *Aegle marmelos* leaf extract, 200 mg/kg BW; *AM*400: *Aegle marmelos* leaf extract 400 mg/kg BW; *AM*600: *Aegle marmelos* leaf extract, 600 mg/kg BW.

A motor activity test was used to measure the frequency of movement around the cage via an infrared beam and the time was recorded for 5 min. This test was used in all groups. Day 0 was the day before scopolamine injection. When the rats were in the frame box, they were explored around, sniffed, climbed, reared, and groomed their bodies. These were normal movements and there was no difference between the groups (Figure 3A). On day 5 after scopolamine injection, we found that the motor activity of the AD group (189 ± 35 times/5 min) did not differ significantly from that of the control group (203 ± 62 times/5 min). However, the motor activity of the *AM*200 group (321.90 ± 60.66 times/5 min) was significantly higher than that of the *AM*400 group (200 ± 27 times/5 min) (Figure 3B). On day 15, there were no significant differences in motor activity among the AD (189 ± 35 times/5 min), *AM*200 (207 ± 56 times/5 min), *AM*400 (153 ± 57 times/5 min), or *AM*600 (154 ± 51 times/5 min) groups (Figure 3C). As a result, scopolamine and *AM* leaf extract had no effect on motor movement in rats experiencing AD-like symptoms.

### 3.3. Effects of the AM Leaf Extract on Memory Impairment

**Figure 4:**
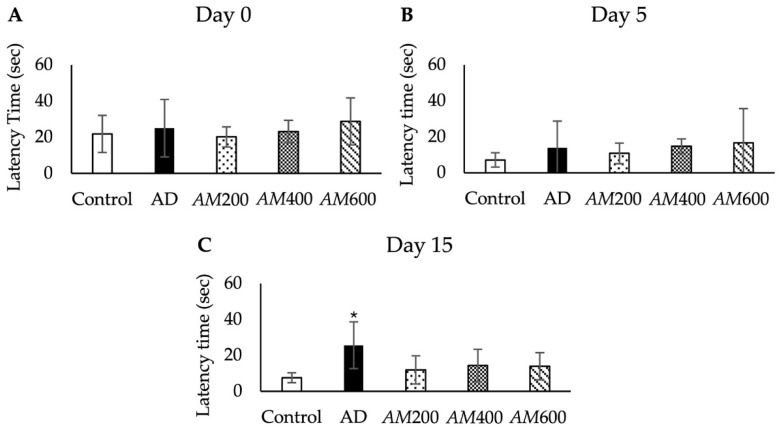
Histogram showing no significant differences between groups in their latency times when finding the hidden platform in the MWM test on day 0 (a day before induction; (**A**)) and day 5 (5 days after the induction; (**B**)). On day 15 (10 days after *AM* treatment; (**C**)), the latency times of the AD group were significantly higher than those of the control group, while those of the *AM*200, *AM*400, and *AM*600 groups were not different from that of the control group. The latency time data (in seconds) are shown as the mean ± SD; *n* = 6. * *p* < 0.05 versus the control group, analyzed via a one-way ANOVA.

The rats in all groups (i.e., control, AD, *AM*200, *AM*400, and *AM*600) were investigated in terms of their latency times when finding the hidden platform in the MWM test on days 0, 5, and 15. The latency times between all groups were not significantly different on day 0 (Figure 4A) nor on day 5 (Figure 4B). However, the latency times of all groups on day 5 tended to show decreases compared with those on day 0. After a week of training from days 6 to 12, the rats in all groups were tested in the final round on day 15. The results showed that the latency times of the AD group (25 ± 13 s) were significantly higher than those of the control group (7 ± 2 s), at *p* < 0.05, while those of the *AM*200, *AM*400, and *AM*600 groups were less than those of the AD group and showed no significant difference from the control group (Figure 4C). As a result, the scopolamine-induced AD-like symptoms caused impairments in the rats’ learning and memory. After treatment with *AM* leaf extract at doses of 200, 400, and 600 mg/kg BW, the rats with AD-like symptoms showed improvements in their cognitive function.

### 3.4. Effects of the AM Leaf Extract on the Density of the Presynaptic Vesicle Proteins in the Axon Terminals of CA1

**Figure 5:**
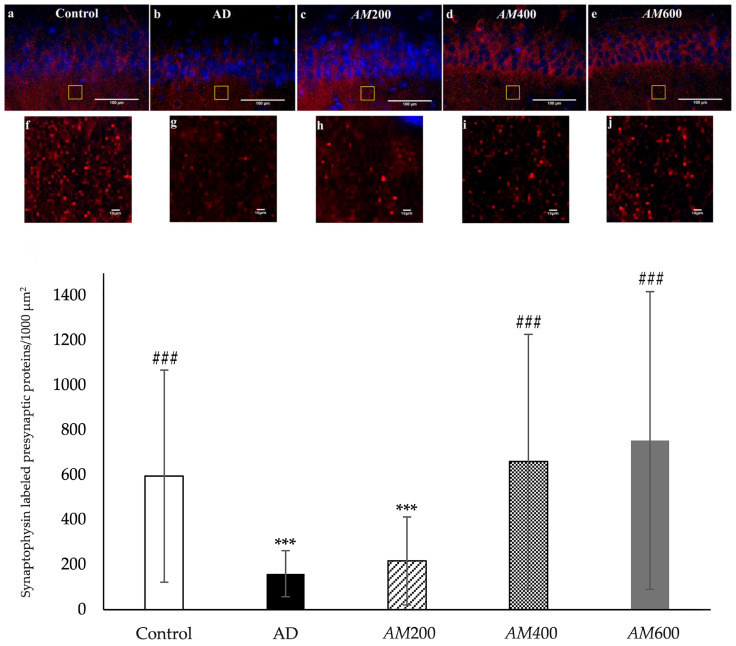
Histograms showing that the synaptophysin density in the CA1 of the *AM*400 and *AM*600 groups significantly increased after *AM* treatment (*n* = 60, ### *p* < 0.001 versus the AD group, analyzed via one-way ANOVA). The synaptophysin density of the AD and *AM*200 groups was significantly lower than that of the control group. The data are shown as the mean ± SD; *n* = 60, *** *p* < 0.001 versus the control group, analyzed via one-way ANOVA). The synaptophysin-immunolabeled presynaptic vesicle proteins (red) and the DAPI-labeled nuclei (blue) in the CA1 of the hippocampi of the control (**a**), AD (**b**), *AM*200 (**c**), *AM*400 (**d**), and *AM*600 (**e**) groups are arranged in a row above the graph. The systemically selected areas in (**a**–**e**) are 1000 µm^2^, used to analyze the synaptophysin density using the ImageJ program, and their magnifications are shown in (**f**–**j**), respectively. Scale bar = 100 µm in (**a**–**e**) and 10 µm in (**f**–**j**).

Synaptophysin was used to label the presynaptic vesicle proteins (PVPs) in the axon terminals at the CA1 area of the hippocampus by immunofluorescence. The proteins are represented as red spots around the nuclei of the pyramidal neurons, which were labeled using DAPI in the CA1 area (Figure 5a). The PVP density was counted per 1000 µm^2^. The results showed that the PVP density of the AD and *AM*200 groups was significantly lower than those of the control group (Figure 5b). However, the rats that received *AM* leaf extract doses of 400 mg/kg and 600 mg/kg had a significantly higher PVP density than the AD group, at *p* < 0.001. Therefore, *AM* leaf extract at medium and high doses could increase the synaptophysin or presynaptic vesicle proteins in the axon terminals of the CA1 of the hippocampus.

### 3.5. Effects of the AM Leaf Extract on the Density of the Dendritic Spines in the CA1

**Figure 6:**
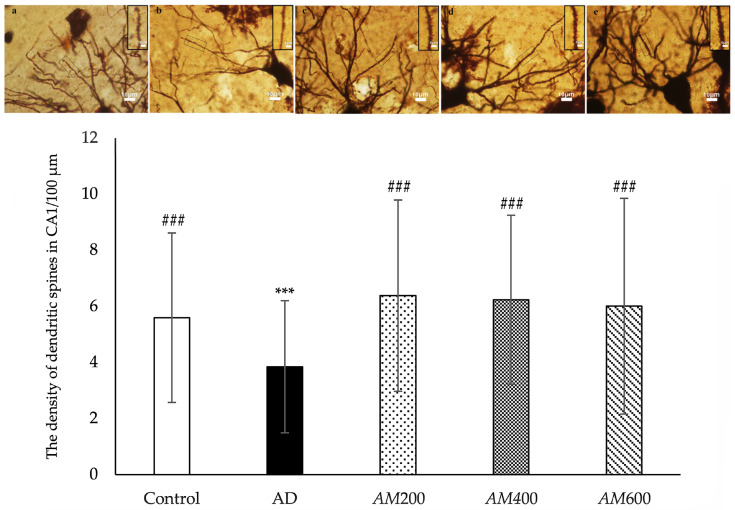
Histogram showing the density of dendritic spines in the CA1 area of the *AM*200, *AM*400, and *AM*600 groups significantly increased after *AM* treatment (*n* = 150; ### *p* < 0.001 versus the AD group, analyzed via one-way ANOVA). The density of dendritic spines in the AD group was significantly lower than that of the control group. The data are shown as the mean ± SD; *n* = 150; *** *p* < 0.001 versus the control group, analyzed via one-way ANOVA. The Golgi-stained dendritic spines of the rats in the control (**a**), AD (**b**), *AM*200 (**c**), *AM*400 (**d**), and *AM*600 (**e**) groups are arranged in a row above the graph. The selected dendrites used for counting the density of each group are magnified, as shown in the inserted panels. The scale bar is 10 µm in all panels. The scale bars in all inserted panels are 5 µm.

Golgi impregnation was used to review the dendritic spines of the pyramidal neurons in the CA1. The dendritic spines on the secondary branch of the dendrites were analyzed to determine the dendritic density as the number of the spines per 100 µm. The results showed that the dendritic spine density of the pyramidal neurons in the CA1 of the AD group (3 ± 2 spines/100 µm) was significantly lower than that of the control group (5 ± 3 spines/100 µm) (Figure 6). The rats that received *AM* leaf extract at doses of 200 mg/kg BW (6.4 ± 3.4 spines/100 µm), 400 mg/kg BW (6.2 ± 3.0 spines/100 µm), and 600 mg/kg (6.2 ± 3.8 spines/100 µm) showed significant increases in dendritic spine density compared to the AD group. As a result, scopolamine caused a reduction in the dendritic spine density. *AM* treatment with low, medium, and high doses could promote and maintain the dendritic spines of the pyramidal neurons in the CA1 regions in rats with AD-like symptoms.

### 3.6. Effect of the AM Leaf Extract on the Histological Structure of the Hippocampus

**Figure 7:**
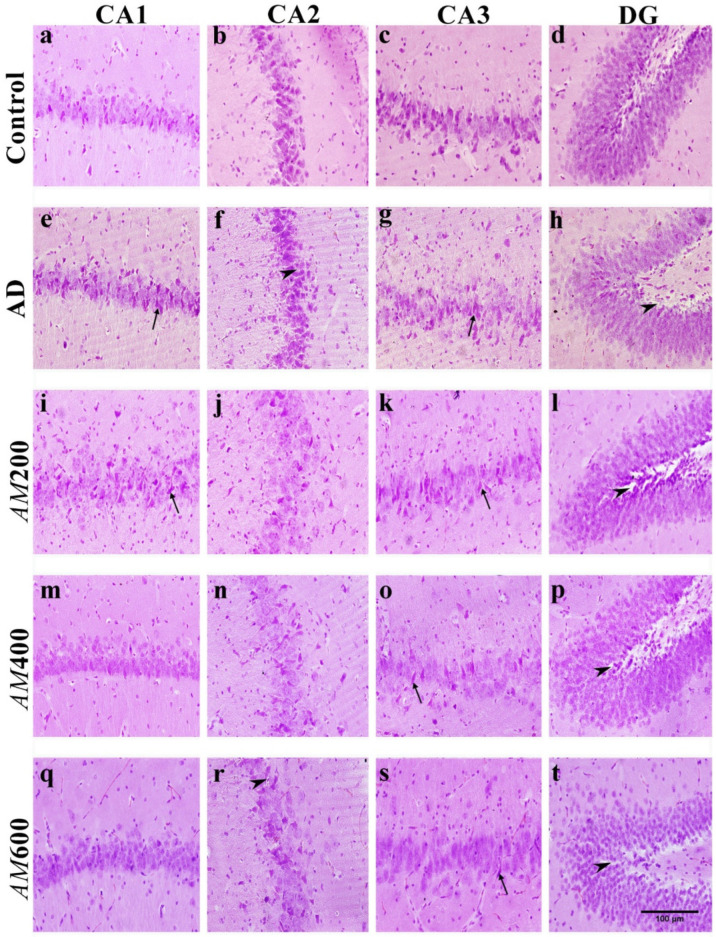
The hematoxylin and eosin-stained histopathological structures of the CA1, CA2, CA3, and dentate gyrus of hippocampi in the AD (**e**–**h**), *AM*200 (**i**–**l**), *AM*400 (**m**–**p**), and *AM*600 (**q**–**t**) groups compared to that of the control (**a**–**d**). AD: Alzheimer’s disease-like symptoms; *AM*200: *Aegle marmelos* leaf extract, 200 mg/kg BW; *AM*400: *Aegle marmelos* leaf extract, 400 mg/kg BW; *AM*600: *Aegle marmelos* leaf extract, 600 mg/kg BW; CA1: Cornu ammonis 1; CA2: Cornu ammonis 2; CA3: Cornu ammonis 3; DG: Dentate gyrus. Scale bars in all panels = 100 µm (arrow head, vacuolation; arrow, shrunken neuron).

The hippocampal formation includes the CA1, CA2, CA3, and DG regions. The main neurons in the CA1–CA3 region of the hippocampus are pyramidal cells and those in the dentate gyrus are granule cells. The histopathological results demonstrated that the animals treated with scopolamine, or the AD group, showed neuronal degeneration with hyperchromatic nuclei and shrinkage of the pyramidal and granule neurons (Figure 7e–h). This resulted in vacuolations in the surrounding neuropil and between neurons compared to the control group. In the *AM*200 group (Figure 7i–l), neurons in most regions of the hippocampus were also shown to be hyperchromatic nuclei and there was a shrinkage of neurons, but not as much as that found in the AD group. In the *AM*400 group (Figure 7m–p), most neurons contained eosinophilic cytoplasm, and the central nucleoli appeared as typical neurons. However, the pyramidal and granule cells of the hippocampi of the *AM*600 group, which received the highest dose, did not show the same degree of recovery of neurons in the CA1 and DG (Figure 7q,t) as the *AM*400 group did (Figure 7m,p). This reveals that *AM* leaf extract at a dose of 400 mg/kg BW may improve the histopathological structures of neurons in the hippocampus.

## 4. Discussion

*AM* leaf extract at doses of 200, 400, and 600 mg/kg BW was used to treat rats with Alzheimer’s disease-like symptoms to investigate their therapeutic properties. Amnesia or cognitive impairment was induced in the rats via scopolamine, a nonselective muscarinic receptor antagonist. The scopolamine dose was 1 mg/kg BW, each day for five days, used to induce Alzheimer’s disease-like symptoms as has been achieved in previous studies [9,11,12,18,32,33]. Memory impairment of the AD groups was not detectable on day 5 post-induction in this study. The symptoms in the AD model might have developed after more than five days following induction via 1 mg/kg BW of scopolamine, as reported previously [34]. Memory deficits caused by the administration of scopolamine are assessed using the MWM test. In this study, the AD model underwent five consecutive days of induction with the same dosage of scopolamine, resulting AD symptoms on day 10 after induction. The latency time to find the submerged platform in the AD group significantly increased compared to the control group in ways shown in previous studies [9,11,12,18,20].

In this study, the motor activities of the AD rats were not different from the control group on days 5 and 15 after the induction of scopolamine. Scopolamine did not affect motor movement, as per the results of a previous study [33]. The acetylcholine receptor in skeletal muscle is a nicotinic receptor that is not affected by nonselective muscarinic receptor antagonists such as scopolamine. This confirms that scopolamine did not affect the skeletal muscles of the rats. However, there was a significant difference in the motor activity on day 5 after scopolamine induction between the *AM*200 and *AM*400 groups, which had not yet received the *AM* extract. These behaviors might have resulted from several factors, such as the drug dose, stress, testing, etc. [7]. Our results showed that the *AM* treatment at all doses did not affect the motor activity of the AD rats.

We treated the rats with AD-like symptoms with *AM* leaf extract at doses of 200, 400, and 600 mg/kg BW. We observed that the rats in all groups demonstrated aggressive behaviors and mood swings and engaged in fighting with other rats. Previous studies have reported that *AM* leaf aqueous extract at a dose of 250 mg/kg reduced testicular sperm count, epididymal sperm count, and motility, as well as caused abnormal sperm count, which is associated with low sex hormones [35]. Testosterone is the principal sex hormone in males that is secreted by Leydig or interstitial cells in the testes, which is a response to anterior pituitary luteinizing hormones [36]. A low level of testosterone in men can cause mood changes such as depression, anxiety, and irritability [36]. The mood changes of the rats in the *AM* groups probably resulted from a decrease in testosterone, as mentioned above. On the contrary, it has been reported that methanol *AM* leaf extract can ameliorate stress disorders such as depression and anxiety [37].

Our H&E staining revealed abnormal histological features in the AD hippocampi, including spaces in the neutrophil surrounding the pyramidal cells in the CA1 and hyper-chromatic nuclei and shrunken neurons, which is consistent with a previous study [38]. We also found that *AM* leaf extract at a dose of 400 mg/kg was the most effective for improving the neuronal architecture of the CA1 in the scopolamine-induced AD models, which is consistent with a previous study [24]. We examined the dendritic spines using the Golgi impregnation method. The number of spines on the secondary dendrites was counted and analyzed to determine the dendritic spine density of the pyramidal cells in the CA1 of the hippocampus, as was carried out in an earlier study [16]. The dendritic spine density in the AD groups was reduced compared to that in the control, which is consistent with previous studies [12]. This reduction in the number of the spines in the AD groups probably caused a change in the neuronal architecture of the hippocampus, resulting in spaces in the neuropil, as shown by H&E staining. In this study, the density of dendritic spines of the secondary dendrites of the pyramidal cells in the CA1 in the *AM*200, *AM*400, and *AM*600 groups increased after induction. Dendritic spines have a significant role in higher brain functioning, including learning and memory. The dendritic spine density depends on the connectivity of the neurons, axon projections to dendritic arbors, number of spines, and dendritic length. Moreover, spine pathology has been studied based on several parameters, including total dendritic length, dendritic diameter, spine area, spine length and spine head, and neck diameter [39]. In this study, scopolamine caused neuronal degeneration or neuronal shrinking, which consequently resulted in a loss of dendritic spines and memory deficits. The regions where axon terminals or presynaptic profiles make contact with dendritic spines or postsynaptic profiles is called a “synapse”.

Synaptophysin is a presynaptic vesicle protein that is particularly associated with AD as part of cognitive functioning. It has been reported that scopolamine significantly decreased the level of synaptophysin compared to the control group [13]. In this study, the density of the synaptophysin-labeled presynaptic vesicle proteins in the axon terminals in the *AM*400 and *AM*600 groups was higher than that in the rats with scopolamine-induced AD-like symptoms, which is consistent with a previous study [12]. Our study showed the relationship between the density of synaptophysin-labeled proteins in the axon terminals, as well as the density of dendritic spines, which is similar to a previous study [12]. However, the synaptophysin density of the *AM*200 group was not significantly higher than that of the AD group, while the density of the dendritic spines was. The previous study reported that both parameters were reduced in the infantile damaged brain, with no correlations occurring between them [40]. The synaptophysin and PSD95 Western blot data need to be examined and the dendritic spines on the primary dendrites need to be quantified, as they were in a previous study [39]. Synaptic loss in the adult hippocampus is directly involved in the memory impairments found in epilepsy, aging, and neurodegenerative diseases [33]. This finding could be applied to enhancing synaptic plasticity and memory in AD.

There are factors related to scopolamine-induced AD, such as oxidative stress and a decreased level of cholinergic neurotransmission [5]. *AM* extract has been reported to contain high total phenolic and total flavonoid contents, which have a correlation with antioxidant activity [22]. The phytochemical compounds in *AM* leaf extract identified in this study included cumin aldehyde, galic acid, eugenol, caffeic acid, flavone, and rutin. Flavone and rutin were found to be at higher levels compared to the others. These two phytochemical compounds were commonly found in previous studies [27]. Flavone is the subclass of flavonoids and rutin is a derivative of flavonoids. Every group of flavonoids has potential as an antioxidant but this is particularly true for flavone [41] and it demonstrates cognitive enhancement, as well [42]. Rutin has been reported as having anti-cholinesterase properties [41]. Even though the other four phytochemical compounds of the *AM* extract in this study were identified in lesser amounts than that of flavone and rutin, they also were thought to have memory enhancement properties. Cumin aldehyde and galic acid improve memory deficits through their antioxidant activities [43,44]. Euginol promoted dendritic growth and complexity of neurons in the hippocampus [45]. Lastly, caffeic acid reversed increases of acetylcholinesterase [46]. We expect that *AM* leaf extract can be an effective alternative treatment for AD. However, to demonstrate the antioxidant and the role of the cholinergic activity of *AM* extract at the molecular level, more studies are needed.

## 5. Conclusions

Treatment with *AM* leaf extract ameliorated the memory deficits in the rats with scopolamine-induced AD-like symptoms, manifested as decreased latency times in a dose-dependent manner. However, the most effective dose was 400 mg/kg BW. These findings occurred as a result of manner in which the *AM* extract promoted axonal transportation of the synaptophysin, the presynaptic vesicle proteins, to the axon terminals of the presynaptic neurons, thereby increasing the dendritic spines of the pyramidal neurons in the CA1 of the hippocampus and reversing the histopathological changes occurring in the hippocampus.

## Data Availability

The original contributions presented in the study are included in the article, further inquiries can be directed to the corresponding author.
